# Disrupted neuronal maturation in Angelman syndrome-derived induced pluripotent stem cells

**DOI:** 10.1038/ncomms15038

**Published:** 2017-04-24

**Authors:** James J. Fink, Tiwanna M. Robinson, Noelle D. Germain, Carissa L. Sirois, Kaitlyn A. Bolduc, Amanda J. Ward, Frank Rigo, Stormy J. Chamberlain, Eric S. Levine

**Affiliations:** 1 Department of Neuroscience, University of Connecticut School of Medicine, 263 Farmington Avenue, Farmington, Connecticut 06030, USA; 2 Department of Genetics and Genome Sciences, University of Connecticut School of Medicine, 263 Farmington Avenue, Farmington, Connecticut 06030, USA; 3 Ionis Pharmaceuticals, Carlsbad, California 92010, USA

## Abstract

Angelman syndrome (AS) is a neurogenetic disorder caused by deletion of the maternally inherited *UBE3A* allele and is characterized by developmental delay, intellectual disability, ataxia, seizures and a happy affect. Here, we explored the underlying pathophysiology using induced pluripotent stem cell-derived neurons from AS patients and unaffected controls. AS-derived neurons showed impaired maturation of resting membrane potential and action potential firing, decreased synaptic activity and reduced synaptic plasticity. These patient-specific differences were mimicked by knocking out *UBE3A* using CRISPR/Cas9 or by knocking down *UBE3A* using antisense oligonucleotides. Importantly, these phenotypes could be rescued by pharmacologically unsilencing paternal *UBE3A* expression. Moreover, selective effects of *UBE3A* disruption at late stages of *in vitro* development suggest that changes in action potential firing and synaptic activity may be secondary to altered resting membrane potential. Our findings provide a cellular phenotype for investigating pathogenic mechanisms underlying AS and identifying novel therapeutic strategies.

## Abstract (web summary)

Angelman syndrome (AS) is characterized by developmental delay and intellectual disability, but the underlying pathophysiology is not well understood. Here the authors use induced pluripotent stem cell-derived neurons from AS patients and find impaired maturation of resting membrane potential and action potential firing, and defects in synaptic activity associated with the disease.

Angelman syndrome (AS) is a neurodevelopmental disorder first described in 1965 by Harry Angelman and is characterized by developmental delay, language impairment, intellectual deficits and oftentimes, seizures^1^,^2^. In addition, AS patients have been referred to as ‘Puppet children' because these patients present with ataxia, happy affect and frequent bouts of laughter^1^,^3^. The prevalence of AS is estimated at 1/15,000 and is caused by the loss of the maternal 15q11–q13 chromosomal region^4^.

The 15q11–13 chromosomal region is regulated by genomic imprinting, an epigenetic phenomenon in which the expression of an allele is determined by the parent of origin. AS results from the loss of function of the *UBE3A* gene, a gene housed within this region. Due to genomic imprinting, *UBE3A* is solely expressed from the maternal allele in neurons. The paternal allele is silenced in these cells by the reciprocal expression of a long, non-coding antisense RNA^3^. Therefore, the loss of the maternal allele of *UBE3A* results in the loss of *UBE3A* mRNA and protein in neurons^5^. The *UBE3A* gene encodes an E3 ubiquitin ligase protein, also known as E6-associated protein, a molecule responsible for tagging target proteins for degradation by the proteasome. The loss of ubiquitin ligase function in the neurons of patients with AS could cause the build-up of UBE3A target proteins that may contribute to disease pathogenesis^6^.

Potential targets of UBE3A identified in mice include synaptic proteins such as EPHEXIN-5 and Arc^7^,^8^, suggesting that deficits in synaptic signalling may be involved in AS pathophysiology. Moreover, the frequent seizure phenotype associated with AS could result from changes in neuronal excitability or disturbances to the balance of excitation and inhibition^9^. Additionally, changes in intrinsic membrane properties and axon initial segment have also been reported in AS mouse models^10^,^11^. These data, in combination with findings of impaired synaptic plasticity, altered dendritic spines, and the cognitive and seizure phenotypes associated with these models^12^,^13^,^14^,^15^,^16^, suggest that neurons from AS patients could have impairments in neuronal excitability and synaptic activity.

The advent of induced pluripotent stem cell (iPSC) technology allows for the generation of patient-specific nervous tissue, which can be used to model a variety of neurological disorders^17^. In this study, we generated iPSC-derived neurons from AS patients and unaffected control subjects to compare the development of intrinsic excitability, action potential (AP) firing and excitatory synaptic activity and synaptic plasticity in these cells. We found that neurons derived from control subjects show an *in vitro* maturation of resting membrane potential (RMP), AP firing and spontaneous synaptic activity. Neurons derived from AS patients were similar to controls at early time points, but showed deficits that were generally apparent by 6–8 weeks *in vitro*. Specifically, AS-derived neurons showed a more depolarized RMP, immature AP firing, decreased spontaneous excitatory synaptic activity and reduced capacity for activity-dependent synaptic plasticity. Importantly, these differences were maintained as late as 20 weeks *in vitro*. These phenotypic differences can be directly attributed to loss of *UBE3A* because these differences were mimicked in control neurons by knocking out *UBE3A* in an isogenic CRISPR-Cas9 gene-edited cell line or knocking down *UBE3A* with antisense oligonucleotides (ASOs) against *UBE3A*, and the phenotype could be rescued in AS neurons by pharmacologically increasing paternal *UBE3A* expression with topoisomerase inhibition. Furthermore, the selective effects of either disrupting *UBE3A* at late stages of *in vitro* development or mimicking the altered RMP during i*n vitro* development suggest that changes in AP firing and synaptic activity may be secondary to altered RMP.

## Results

### Characterization of AS and control cultures

**Figure 1:**
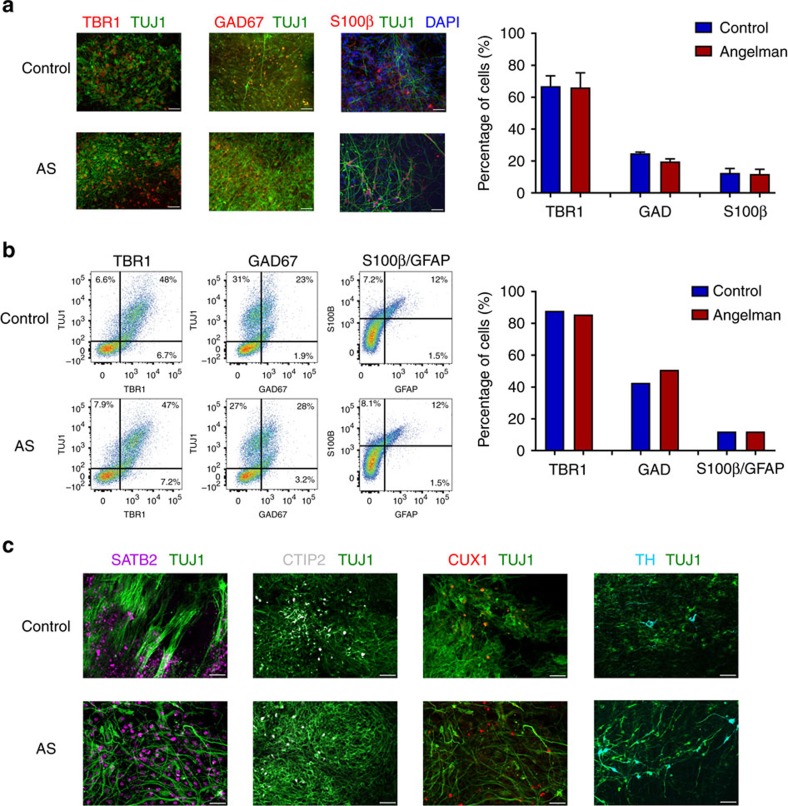
Characterization of cell types in AS and control iPSC-derived cultures. (**a**) Left: Immunocytochemical staining for TBR1/TUJ1 and GAD67/TUJ1, and S100β/DAPI, in control and AS-derived cultures. Scale bar, 50 μm. Right: Group data for expression of TBR1-positive and GAD65/67-positive neurons graphed as a percentage of TUJ1-positive neurons at 5 weeks *in vitro*, and expression of S100β-positive neurons graphed as a percentage of DAPI-positive cells at 17 weeks *in vitro*. *n*≥4 coverslips per condition. (**b**) Left, flow cytometry graphs for TBR1/TUJ1, GAD67/TUJ1 and S100β/GFAP at 16–18 weeks *in vitro*. Right, quantification of TBR1-positive and GAD67-positive neurons as a percentage of TUJ1-positive neurons, and S100β/GFAP-positive neurons as a percentage of DAPI-positive cells. (**c**) Immunocytochemical staining for SATB2, CTIP2, CUX1 and TH in control and AS-derived cultures at 10–22 weeks *in vitro*. For each cell marker, matching AS and control experiments were done at similar ages. Scale bar, 50 μm. Error bars represent mean ±s.e.m.

Cells were plated via manual dissociation of neurosphere structures following the generation of embryoid body-like aggregates (Supplementary Fig. 1d). We used a differentiation protocol that, in the absence of specific morphogens, leads to a default generation of forebrain neurons^18^,^19^,^20^. Similar protocols have been used to generate cortical neurons and functional synaptic networks^21^,^22^. To determine whether the cellular composition of iPSC-derived cultures was affected by the AS genotype, we characterized neuronal cultures using immunocytochemistry and flow cytometry. For immunocytochemical experiments, cultures derived from AS and control subjects were fixed at ≥5 weeks *in vitro* and stained for TUJ1 (β-tubulin), a neuron-specific microtubule protein, TBR1, a transcription factor specific for glutamatergic neurons and GAD65/67 or GAD67, markers for GABAergic neurons. There were no significant differences between control and AS cultures in the proportion of glutamatergic neurons (Fig. 1a; control: 66.9±6.4%; AS: 66.1±9.1%) or GABAergic neurons (Fig. 1a; control: 24.8±0.7%; AS: 19.7±1.6%). We also determined the percentage of astrocytes present in these cultures by staining for the nuclear marker 4,6-diamidino-2-phenylindole (DAPI) and the mature astrocyte marker S100β. At 17 weeks *in vitro*, the proportion of DAPI-positive cells that stained positive for S100β was not significantly different for AS and control cultures (Fig. 1a; control: 12.6±2.7%; AS: 11.9±2.8%).

The similarity in cell composition between AS and controls was confirmed using flow cytometry for these same markers. As shown in Fig. 1b, the proportions of TBR1, GAD67 and S100β/GFAP cells were similar to that seen by immunocytochemistry, and again was not different based on genotype. We also found that cells in both AS and control cultures expressed markers for upper cortical layers (CUX1 and SATB2) and deep layers (CTIP2), as well as a population of tyrosine hydroxylase-positive cells (Fig. 1c). These markers were expressed at similar levels in control and AS cultures, and this was confirmed by flow cytometry (Supplementary Fig. 2).

### Depolarized resting membrane potential in AS neurons

Patch-clamp recordings were conducted on nine iPSC lines derived from three AS patients and four control subjects: two clones each from one AS patient and one control, and single clones from the remaining subjects. In addition, a *UBE3A* knockout (KO) line was generated from one of the control lines using CRISPR/Cas9-mediated gene editing (see Methods). All recorded cells underwent current clamp protocols, for determining RMP and AP firing, as well as voltage clamp protocols, for monitoring inward and outward currents and synaptic currents. A developmental time course was generated by recording weekly from the time of plating (3 weeks post initiation of differentiation) through week 20 of *in vitro* development. Recordings were obtained from at least 15 cells per coverslip and 1–5 coverslips were used per week per genotype.

**Figure 2:**
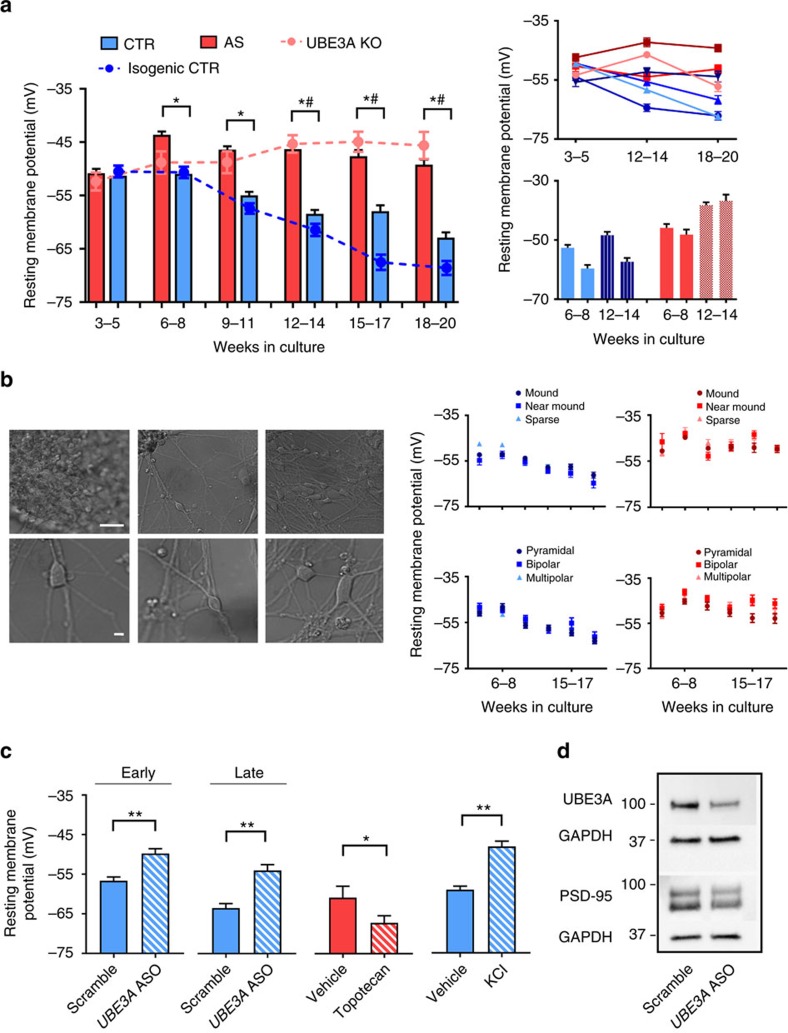
RMP of iPSC-derived neurons. (**a**) Left: Group data for RMP of control (CTR; 4 subjects; *n*>130 at each time point), AS (3 subjects; *n*>150 at each time point) and *UBE3A* KO (UKO; *n*≥45 at each time point) during development (two-way analysis of variance (ANOVA), *P*<0.0001, *differences between control and AS, ^#^differences between control and UKO, Bonferroni *post hoc* test, *P*<0.0001). Upper right: RMP for neurons derived from individual control and AS subjects. For each time bin, *n*≥30 for all lines. Lower right: RMP for neurons derived from two separate iPSC clones from a single control patient (solid and hatched blue bars) and a single AS patient (solid and hatched red bars). For each time bin, *n*≥45 for each clone. (**b**) Left, top row: Classification of culture location (from left to right): mound, near mound and sparse. Scale bar, 25 μm. Left, bottom row: Classification of cell shape (from left to right): pyramidal, bipolar and multipolar. Scale bar, 10 μm. Right, top: Group RMP data from control and AS neurons separated by location. Right, bottom: Group RMP data from AS patient neurons separated by cell shape. For controls, each symbol represents data from four subjects. For AS, each symbol represents data from three subjects (*n*>40 for both genotypes for all morphologies/locations at every time point). (**c**) RMP for (left to right) control neurons treated with *UBE3A* ASOs at 6 weeks in culture (6 coverslips per condition, 15 cells per coverslip), control neurons treated with *UBE3A* ASOs at 18 weeks in culture (4 coverslips per condition, 15 cells per coverslip), AS neurons treated with 1 μM topotecan (2–4 coverslips per condition, 15 cells per coverslip) and control neurons treated with 10 mM KCl from four control subjects (1 coverslip, 15 cells per coverslip for each control line). *P<0.05, **P<0.0001, Student's *t*-test. (**d**) Western blot of UBE3A (100 kD), PSD-95 (95 kD) and GAPDH (37 kDa) from a control culture treated at 6 weeks with 2.5 μM *UBE3A* ASOs. Error bars represent mean ±s.e.m.

As shown in Fig. 2a, the mean RMP of control neurons (from four subjects) and AS-derived neurons (from three patients) was similar at 3–5 weeks *in vitro*. However, the RMP of control neurons showed a subsequent hyperpolarizing shift during development that was not seen in AS-derived neurons. RMP in AS neurons was significantly more depolarized than control neurons over 20 weeks in culture (Fig. 2a). Results from the individual subjects are shown in the upper right panel of Fig. 2a. Similar developmental trends in RMP were observed when comparing neurons derived from two clonal cell lines derived from a single control patient, and two clones from a single AS patient (Fig. 2a, lower right). Specifically, the two clonal lines derived from the control patient showed a developmental shift in RMP towards more hyperpolarized potentials, which was absent in the two clonal lines derived from the AS patient, suggesting that genotypic differences are not likely due to clonal variability. Detailed week-by-week analysis for individual patients and genotype groups is shown in Supplementary Fig. 3a,b.

Due to the heterogeneity of iPSC-derived neurons in culture, we noted the shape and location of each recorded cell by visual inspection to investigate whether phenotypic differences were specific to a particular cell morphology. Cells were categorized according to three basic shapes—pyramidal, bipolar or multipolar (Fig. 2b). In addition, cells were classified according to location within the culture: mound, near mound and sparse (Fig. 2b). Mound cells were those located on the initial clusters of cells generated by neurosphere dissociation, near mound represents cells that migrated away from the mound but still extended processes into the dense mound structures and sparse represents groups of cells that were in areas devoid of mounds. As shown in the right panel of Fig. 2b, the hyperpolarizing shift in RMP of control cells during development, and the lack of this developmental change in AS neurons, was seen uniformly across all cell morphologies and locations.

We next examined the specific role of *UBE3A* loss in the RMP phenotype. Two of the AS patient lines have a large deletion of chromosome 15q11–q13 encompassing many genes, as is seen in the majority of patients with AS. The third line was derived from a patient with AS caused by a 2 bp deletion in *UBE3A* that results in loss of the protein. This patient line shows a similar RMP phenotype to the large deletion lines (Fig. 2a), suggesting that loss of *UBE3A* alone is the cause of the genotypic difference in RMP observed in our AS lines. To further explore the causative role of *UBE3A* loss, CRISPR/Cas9 gene editing was used to target both maternal and paternal *UBE3A* alleles in a control line to generate an isogenic *UBE3A* KO line, which resulted in almost total loss of *UBE3A* protein (Supplementary Fig. 3c). Similar to the AS patient lines, neurons derived from the *UBE3A* KO line also showed a more depolarized RMP throughout development compared to both the grouped control data and the isogenic control line (Fig. 2a), further indicating that loss of *UBE3A* alone is responsible for the RMP phenotype observed in the AS patient lines.

Since KO of *UBE3A* results in loss of UBE3A protein throughout the entire time course of iPSC-derived neuronal development, we next wanted to know whether acute loss of *UBE3A* could also induce the AS phenotype. Treatment of control neurons at 6 weeks in culture (early) for 72 h with ASOs specific for *UBE3A* resulted in ∼60% reduction *UBE3A* mRNA expression (Supplementary Fig. 3e) and UBE3A protein (Fig. 2d), with no change in *GABARB3*, a gene also located in the chromosome 15q11–q13 region. Three to six weeks after ASO treatment, we recorded ASO and scramble-treated neurons and observed a significant depolarization in RMP in the ASO-treated neurons (Fig. 2c), suggesting that acute knockdown of *UBE3A* at early stages of *in vitro* development is sufficient to produce the AS phenotype. Similarly, knockdown of *UBE3A* by ASO treatment at 18 weeks in culture (late) also produced a significant depolarization in RMP in the ASO-treated neurons (Fig. 2c). There was no difference in the knockdown of *UBE3A* by ASO between early and late treated cells (Supplementary Fig. 3e). As ASOs may have off-target effects, experiments were conducted using two different ASOs against *UBE3A*, which showed similar knockdown of *UBE3A* (Supplementary Fig. 3e). There was also no significant difference between high and low dose (Supplementary Fig. 3e), thus all ASO data were combined. ASO treatment of AS-derived neurons, which also acts as a control for off-target effects, showed no change in RMP (Supplementary Fig. 3d), consistent with the lack of *UBE3A* in AS neurons. These data suggest that knockdown of *UBE3A* alone can cause a depolarization of RMP independent of neuron maturity.

Finally, due to neuron-specific genomic imprinting, the paternal copy of *UBE3A*, though silenced, is still present in AS-derived neurons, and therefore is an attractive therapeutic target for restoring endogenous *UBE3A* to normal levels. It has been shown that treatment of an AS model mouse with the topoisomerase inhibitor topotecan can restore some *UBE3A* expression from the paternal allele, most likely by reducing the expression of the antisense that normally silences paternal *UBE3A*^23^. Treatment of AS-derived neurons with 1 μM topotecan for 3–6 weeks resulted in ∼50% increase in *UBE3A* mRNA expression (Supplementary Fig. 3e) and a significant shift to a more hyperpolarized RMP (Fig. 2c). Together, these data suggest that restoring UBE3A protein levels by unsilencing paternal *UBE3A* can rescue the AS RMP phenotype.

### Impaired AP firing in AS neurons

**Figure 3:**
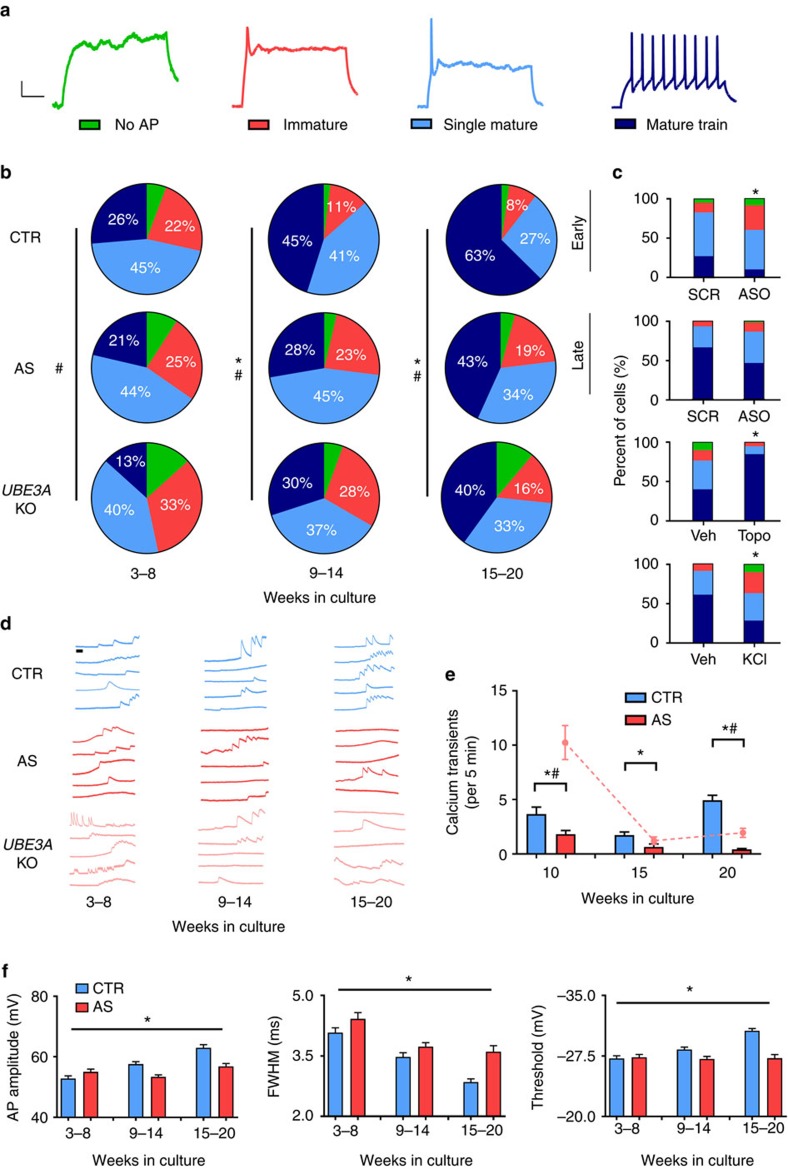
Characterization of AP firing. (**a**) Example traces representing four AP firing patterns used for characterization. Scale bar, 20 mV, 100 ms (**b**) Distribution of AP firing patterns for control (CTR; 4 subjects; *n*>250 at each time point), AS (3 subjects; *n*>250 at each time point) and *UBE3A* KO (1 line; *n*>40 at each time point) neurons at three developmental time bins. *P<0.0001 for differences between control and AS; *χ*^2^ test. ^#^*P*<0.0001 for differences between control and *UBE3A* KO; *χ* test. (**c**) AP firing distributions for (top to bottom) control neurons treated with *UBE3A* ASOs at 6 weeks in culture (7 coverslips, 15 cells per coverslip), control neurons treated with *UBE3A* ASOs at 18 weeks in culture (4 coverslips, 15 cells per coverslip), AS neurons treated with 1 μM topotecan (2–4 coverslips, 15 cells per coverslip), KCl (10 mM) treated control neurons from four control subjects (*n*=15 cells per coverslip for 1 coverslip for both vehicle and KCl for each control line). *P<0.0001, *χ*^2^ test. (**d**) Example calcium imaging traces of spontaneous activity from control, AS and *UBE3A* KO cultures; scale bar, 30 s. (**e**) Quantification of number of calcium transient for control, AS and *UBE3A* KO cultures (*n*>60 at each time point for all three genotypes. *P<0.05 (Student's *t*-test) for differences between control and AS. ^#^*P*<0.05 (Student's *t*-test) for differences between control and *UBE3A* KO. (**f**) AP amplitude (left), FWHM (middle) and AP threshold (right) for control and AS cultures at three time points (*n*>250 for both genotypes at each time point). *P<0.0001 for significant differences between control and AS (two-way analysis of variance (ANOVA)). Error bars represent mean ±s.e.m.

AP firing in response to intracellular current injection was categorized as either No AP, Immature, Single Mature or Mature Train (see example traces in Fig. 3a). APs were characterized as mature if AP amplitude was >35 mV and full-width at half-maximal amplitude (FWHM) was <5.5 ms. An immature AP was one that failed to meet these criteria. A mature train was defined as having three or more mature APs during the 500 ms depolarizing step. At 3–8 weeks in culture, neurons from controls and AS patients showed similar distributions (Fig. 3b). Week-by-week data for each group are shown in Supplementary Fig. 4g. It is interesting to note that even immediately after plating (3 weeks), 50–60% of cells from both control and AS-derived cultures had mature firing. At later time points, however, there were significant genotype differences. Control neurons showed a significant developmental shift to a larger proportion of cells expressing mature AP firing, reaching 90% of cells at 20 weeks in culture. AS-derived neurons failed to show similar developmental maturation, resulting in a lower proportion of cells that fired AP trains and a higher proportion of immature APs in AS neurons (Fig. 3b). A similar lack of maturation of AP firing was observed in the isogenic *UBE3A* KO line compared to control neurons (Fig. 3b).

Similar to the RMP results, treatment of control neurons with *UBE3A* ASO at early stages of *in vitro* development resulted in a significantly more immature distribution of AP firing compared to scramble-treated neurons (Fig. 3c). However, unlike our observations with RMP, treatment of control neurons with *UBE3A* ASO at late stages of *in vitro* development failed to cause a shift in the AP firing distribution (Fig. 3c). AS neurons treated with ASO showed no changes in the percent of neurons showing mature firing (Supplementary Fig. 4h). It has previously been shown that depolarization of iPSC-derived neurons with KCl can result in a shift to more immature AP firing^24^. This led us to wonder if the immature firing observed in AS patient neurons and *UBE3A* KO neurons are secondary to the depolarized RMP observed in these cells. To answer this question, control neurons were grown in media supplemented with a higher concentration of KCl (10 mM) for 20 weeks of *in vitro* development. Similar to AS patient neurons and *UBE3A* KO neurons, KCl treatment of control neurons (without loss of *UBE3A*) caused a shift to more immature firing at late stages of *in vitro* development (Fig. 3c). Interestingly, depolarization with KCl also resulted in an intrinsic depolarization of control neurons (Fig. 2c). Treatment of AS neurons with topotecan to restore *UBE3A* levels resulted in a complete rescue of AP firing to control levels (Fig. 3c).

To examine spontaneous AP firing in unperturbed cells, we used calcium imaging to indirectly monitor activity. Cells were incubated in 10 μM Fluo-4AM for 1 h at 37 °C and then imaged at 100 Hz. three coverslips/condition/time point were used at 10, 15 and 20 weeks *in vitro* as an early, middle and late developmental period. Example traces for control, AS and *UBE3A* KO experiments are shown in Fig. 3d. Calcium transients were blocked by tetrodotoxin (1 μM; data not shown). Across all three time points, AS neurons showed significantly fewer calcium transients during the imaging period compared with control neurons (Fig. 3e). *UBE3A* KO neurons also showed significantly fewer calcium transients than controls at the most mature time point (Fig. 3e) but were not significantly different at the middle time point. Interestingly, *UBE3A* KO neurons displayed significantly more calcium transients than both AS and control cultures at the earliest time point.

We next examined properties of individual APs. Significant differences were observed in AP amplitude, FWHM and spike threshold between AS and control neurons (Fig. 3f). AP amplitude, threshold and FWHM all showed significant developmental shifts for control neurons which is expected given the maturation of AP firing observed in Fig. 3b. However, AS neurons did not show a significant change across development for either AP amplitude or FWHM, which might explain their more immature AP firing observed throughout *in vitro* development (Fig. 3b). Similar results were obtained when comparing individual patient lines and grouped lines for the full week-by-week analysis of these features (Supplementary Fig. 4a,b). These trends were also uniform across cell morphology (Supplementary Fig. 4c,d) and location (Supplementary Fig. 4e,f).

### Comparison of intrinsic membrane currents

Whole-cell voltage clamp recordings were used to examine intrinsic voltage-gated conductances. Supplementary Figure 5a depicts example inward and outward current traces from a control neuron at week 12 *in vitro*. Inward currents were completely blocked by the sodium channel blocker tetrodotoxin (500 nM). Group data for the current–voltage relationship for inward and sustained outward currents is shown in Supplementary Fig. 5b. Comparison of maximum inward and outward current densities, normalized to cell capacitance (Supplementary Fig. 5c), revealed significant genotype differences in inward current density as well as sustained and transient outward current densities (Supplementary Fig. 5d–f). Interestingly, transient outward current showed a significant increase throughout *in vitro* development in both control and AS-derived neurons (Supplementary Fig. 5f), consistent with the developmental decrease in AP width, but remained significantly less in AS neurons compared to controls throughout development, which may account for the increased FWHM and more immature firing observed in AS neurons. The full week-by-week analysis for intrinsic membrane currents for individual patients and genotype groups is shown in Supplementary Fig. 6a,b. These trends were also uniform when analysed by neuronal morphology (Supplementary Fig. 6c,d) or location (Supplementary Fig. 6e,f). Finally, we compared input resistance of neurons from both genotypes during weeks 6 to 12 of *in vitro* development. No significant difference in input resistance was observed between AS and control neurons (control: 2.82±0.12 GΩ, *n*=166; AS: 2.52±0.11 GΩ, *n*=174). Capacitance of AS neurons compared to control neurons was not significantly different (Supplementary Fig. 5c).

### Spontaneous synaptic activity and synaptic plasticity

**Figure 4:**
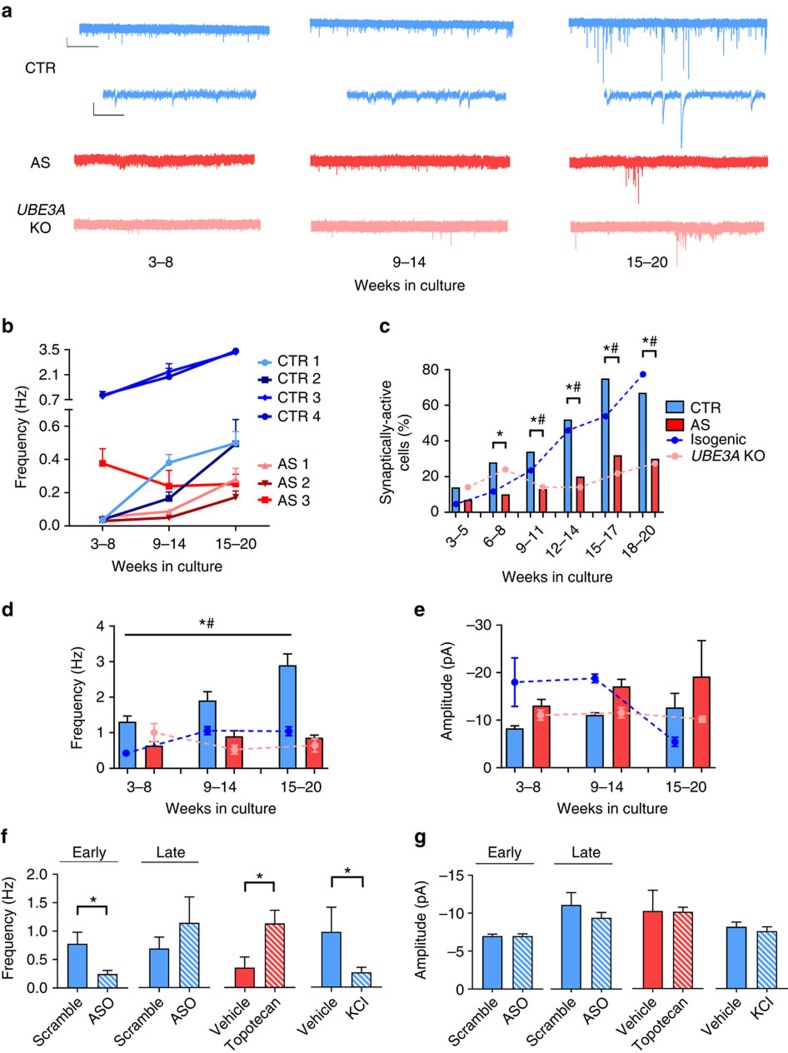
Development of spontaneous excitatory synaptic activity. (**a**) Example traces of spontaneous excitatory synaptic currents from control (CTR), AS and *UBE3A* KO neurons at 3–8, 9–14 and 15–20 weeks in culture. Scale bar for top CTR, AS and *UBE3A* KO, 10 pA, 5 s. Scale bar for bottom CTR traces, 10 pA, 100 ms. (**b**) Frequency of spontaneous synaptic currents for neurons derived from all CTR and AS subjects during development (for both groups, *n*>40 cells at each time point). (**c**) Percent of synaptically active neurons (event frequency >0.2 Hz) derived from CTR (4 subjects, *n*>60 cells at every time point), AS subjects (3 subjects, *n*>40 cells at every time point) and *UBE3A* KO (1 line; *n*>40 cells at every time point) during development. *P<0.05 for significant differences between CTR and AS. ^#^*P*<0.05 for significant differences between CTR and *UBE3A* KO line (*χ*^2^ test). (**d**) Mean frequency of spontaneous synaptic events for active neurons derived from CTR (4 subjects, *n*>40 cells at every time point), AS subjects (3 subjects, *n*>35 cells at every time point) and *UBE3A* KO (1 line; *n*>15 cells at every time point). (**e**) Mean amplitude of spontaneous synaptic events for neurons plotted in **d**. *P<0.05 for differences between CTR and AS. ^#^*P*<0.05 for differences between CTR and *UBE3A* KO line (two-way analysis of variance (ANOVA)). (**f**) Synaptic frequency and (**g**) amplitude for (left to right) CTR neurons treated with *UBE3A* ASOs at 6 weeks in culture (6 coverslips per condition, 15 cells per coverslip), CTR neurons treated with *UBE3A* ASOs at 18 weeks in culture (4 coverslips per condition, 15 cells per coverslip), AS neurons treated with 1 μM topotecan (2–4 coverslips per condition, 15 cells per coverslip), 10 mM KCl-treated CTR neurons from 4 CTR subjects (1 coverslip for both vehicle and KCl for each CTR line, 15 cells per coverslip). *P<0.05, Student's *t*-test. Error bars represent mean ±s.e.m.

The ability to form functional synaptic networks and express activity-dependent synaptic plasticity is a hallmark of neuronal maturation. Deficits in synaptic signalling and plasticity are seen in animal models of AS (reviewed in ref. 2) and are thought to underlie aspects of the AS phenotype in patients. We therefore examined spontaneous excitatory synaptic transmission in neurons derived from AS patients and control subjects (see example traces in Fig. 4a). Excitatory synaptic currents were blocked by the AMPA (α-amino-3-hydroxy-5-methyl-4-isoxazole propionic acid) receptor antagonist DNQX (10 μM; data not shown). Control neurons showed markedly greater developmental increases in the frequency of synaptic events compared to neurons derived from AS patients (Fig. 4b). In addition, as shown in Fig. 4c, the percentage of synaptically active cells (event frequency >0.2 Hz), although not different across genotype at early time points, was significantly higher in control compared to AS neurons later in development, a difference that was maintained up to 20 weeks in culture. Neurons derived from the isogenic *UBE3A* KO line also showed a significantly lower proportion of synaptically active neurons throughout development. These differences were uniform across all cell morphologies and locations (Supplementary Fig. 7a–d). In addition, as shown in Fig. 4d, the event frequency in synaptically active cells was significantly lower in AS-derived neurons compared to control neurons throughout development. There were no significant differences in amplitude of spontaneous synaptic events in AS neurons compared to control neurons (Fig. 4e), but there was a trend towards larger amplitudes in AS neurons. Overall, the impairments in RMP, AP and synaptic development in AS neurons could be related to cell death after prolonged time in culture. To test this, we performed a lactase dehydrogenase (LDH) assay on control, AS and *UBE3A* KO cultures at 15–20 weeks *in vitro*, which showed no significant differences in LDH activity (Supplementary Fig. 7f).

Treating control neurons with *UBE3A* ASO at early stages of *in vitro* development resulted in a significant decrease in the frequency of synaptic events recorded 3–6 weeks later (Fig. 4f). Acute knockdown of *UBE3A* at a later stage of development did not significantly affect the frequency of synaptic activity (Fig. 4f). Similar to AP firing, the lack of effect of late ASO treatment suggests that the changes in synaptic activity in AS neurons and *UBE3A* KO neurons may be secondary to the depolarized RMP. In support of this, treatment of control neurons throughout development with KCl resulted in a significant decrease in synaptic frequency compared to vehicle-treated control neurons (Fig. 4f). Treatment of AS neurons with *UBE3A* ASO had no effect on synaptic frequency or amplitude (Supplementary Fig. 7e). Finally, AS neurons treated for 3–6 weeks with topotecan, which has been shown to increase *UBE3A* by activating the paternal allele, resulted in a significant increase in synaptic frequency (Fig. 4f). In all cases (early or late ASO, topotecan, KCl) there were no observed changes in synaptic event amplitude (Fig. 4g). We also performed an LDH assay on vehicle- and KCl-treated cultures at 20+ weeks *in vitro* to confirm that the observed differences with KCl treatment were not due to increases in cell death. No significant difference in LDH activity was observed between vehicle- and KCl-treated cultures (Supplementary Fig. 7f).

**Figure 5:**
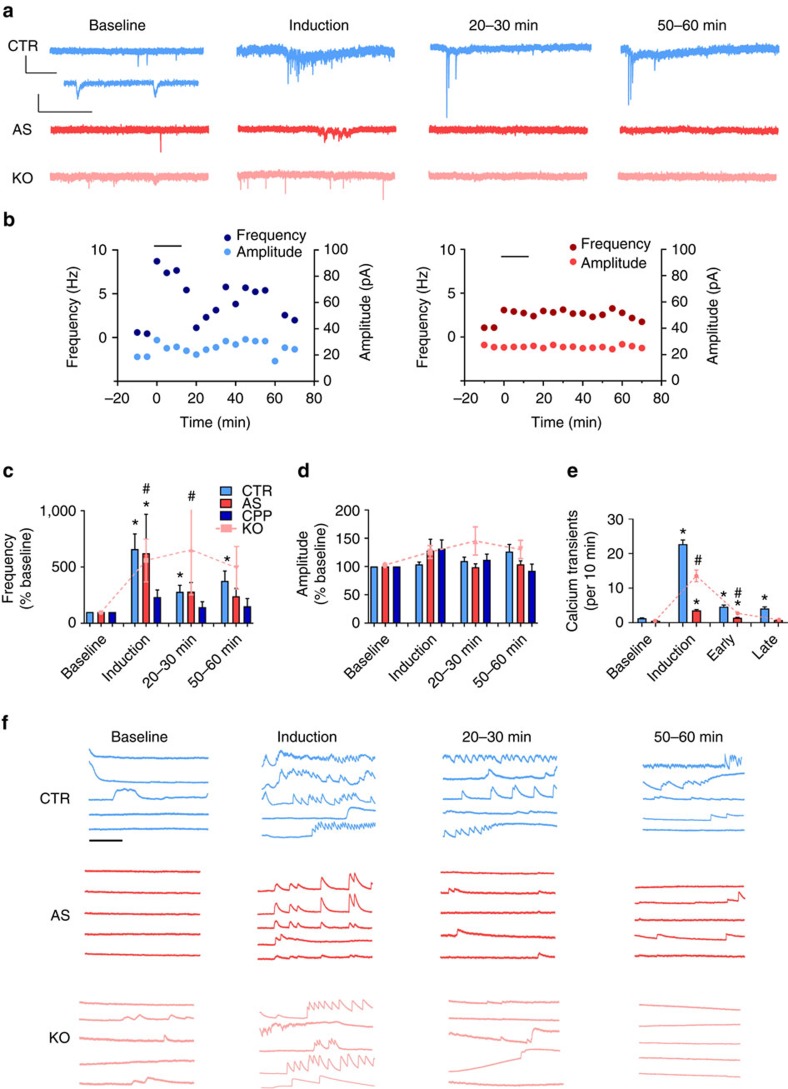
Synaptic plasticity in iPSC-derived neurons. (**a**) Example traces of spontaneous excitatory synaptic currents from control (CTR; top), AS (middle) and *UBE3A* KO (bottom) neurons at (left to right) baseline, induction, 20–30 min post induction, and 50–60 min postinduction (see Methods for details). Scale bar for top CTR, AS and *UBE3A* KO, 20 pA, 1 s. Scale bar for bottom CTR trace, 10 pA, 200 ms. (**b**) Example time courses for frequency and amplitude of spontaneous synaptic currents from an individual neuron from (left) a CTR subject and (right) an AS patient. Plasticity induction period indicated by black bar. (**c**) Individual data for CTR and AS-derived neurons (12–25 weeks in culture) showing frequency of spontaneous synaptic currents during plasticity induction and 20–30 and 50–60 min post induction. *P<0.05, indicates difference for CTR or AS; ^#^for *UBE3A* KO (Student's *t*-test). (**c**,**d**) Group data for CTR (*n*=30), AS-derived (*n*=20) *UBE3A* KO (*n*=9) and +CPP (*n*=8) neurons showing (**c**) mean frequency and (**d**) mean amplitude of spontaneous synaptic currents during plasticity induction and 20–30 and 50–60 min post induction. (**e**) Quantification of frequency of calcium transients from CTR, AS and *UBE3A* KO lines pictured in **f** (*n*>240 for CTR and AS; *n*>80 for *UBE3A* KO). *P<0.05, indicates difference for CTR or AS; ^#^for *UBE3A* KO (Student's *t*-test). Early: 20–30 min post induction; late: 45+ min post induction. (**f**) Example traces of calcium imaging during same protocol as above from (top) CTR, (middle) AS and (bottom) *UBE3A* KO cultures. Scale bar, 3 min. Error bars represent mean ±s.e.m.

To test the ability of iPSC-derived neurons to undergo activity-dependent synaptic plasticity, we used a chemically induced long-term potentiation (LTP) protocol that stably increases intracellular cAMP levels and enhances NMDA receptor activation^25^. Following baseline measurements, cells were exposed to the induction cocktail for 15 min, followed by washout to normal bath solution. Example traces from control, AS and *UBE3A* KO neurons are shown in Fig. 5a. Time courses for the synaptic event frequency and amplitude for a control and an AS-derived neuron are shown in Fig. 5b. Overall, there was a significant increase in frequency of spontaneous currents during the induction period for both control and AS-derived neurons (Fig. 5c). A significant increase in event frequency was still present at both 20–30 min and 50–60 min post induction for control neurons. AS neurons, however, failed to show significant increases in frequency at either 20–30 or 50–60 min post induction. Neurons from the *UBE3A* KO line also showed a significant increase in frequency during the induction, but failed to maintain this increase at 50–60 min post induction, although they did show a significant increase at 20–30 min. There was no difference in event amplitude by time or genotype (Fig. 5d). Plasticity was dependent on NMDA receptor activation as it was completely blocked in the presence of the NMDA receptor antagonist CPP (3 μM; Fig. 5c,d).

To examine whether this plasticity was also observed at the level of AP firing, we used population calcium imaging. Example traces from control, AS and *UBE3A* KO neurons at baseline, during induction and 20–30 min and 45+ min post induction are shown in Fig. 5f. Interestingly, synchronous activity across cells was sometimes observed. Control neurons showed a large increase in the number of calcium transients during induction that was maintained for up to 45+ min post induction (Fig. 5e). Although AS and *UBE3A* KO neurons showed significant increases in the number of transients during induction and 20–30 min post induction, they failed to maintain this increase at 45+ min post induction.

## Discussion

AS is a genomic imprinting disorder caused by loss of function of the maternally inherited allele of the E3 ubiquitin ligase, *UBE3A*. We have previously shown that normal imprinting of *UBE3A* is established during neuronal differentiation of iPSCs derived from AS and control subjects, and UBE3A protein expression is largely absent in neurons derived from AS iPSCs^26^. In the present study, we examined genotypic differences in *in vitro* development and maturation in iPSC-derived neurons from AS patients and unaffected controls, from the time of initial plating up to week 20 post differentiation, considerably longer than previous examinations of iPSC-derived neurons. Cells were also classified by shape and by location with respect to their proximity to cell mounds. For both genotypes, the majority of neurons were TBR1-expressing, presumably glutamatergic, neurons, while GAD65/67-expressing GABAergic neurons constituted the other major cell population, and there were no differences between AS and control cultures in the proportion of glutamatergic neurons, GABAergic neurons or astrocytes.

Neurons from AS patients had several significant differences compared to neurons derived from unaffected controls. Specifically, AS-derived neurons displayed a more depolarized RMP, a reduction in the proportion of cells that fired mature APs, a reduction in excitatory synaptic activity and a deficit in activity-dependent synaptic plasticity. These differences were seen in cells from two AS patients with a large deletion, as well as a patient with a mutation in *UBE3A* alone. Interestingly, these differences were generally not present at the earliest stages of development, but reflected a failure of AS-derived neurons to undergo the developmental maturation observed in control neurons. Notably, some aspects of maturation, for example, synaptic activity, showed continued development up to 20 weeks in culture reaching levels of activity greater than typically reported in other studies of iPSC-derived neurons. This highlights the importance of allowing for prolonged maturation in these cultures. Additionally, these genotypic differences were consistent across different neuronal morphologies and locations, suggesting that the phenotype is shared by multiple cell types.

Several lines of evidence support the idea that these genotypic changes are attributed directly to loss of *UBE3A*. First, cells derived from the patient with a *UBE3A* mutation had the same cellular phenotype as cells derived from the large deletion patients. Second, knocking out *UBE3A* in an isogenic CRISPR-Cas9 gene-edited cell line replicated the phenotype. Third, knocking down *UBE3A* with an ASO produced the same cellular phenotype. Moreover, we were able to rescue this phenotype by unsilencing *UBE3A* expression from the paternal allele with the topoisomerase inhibitor topotecan. Interestingly, early ASO knockdown of *UBE3A* affected RMP, AP firing and synaptic activity, whereas late knockdown only affected RMP, suggesting that changes in RMP may be directly related to *UBE3A* loss and AP and synaptic changes may be secondary effects. In support of this idea, we found that control neurons, grown under slightly depolarizing conditions to mimic AS neurons, showed similar disruptions in AP firing and synaptic activity. Findings from a recently published study of iPSC-derived neurons further support this argument^27^. This study, which classified iPSC-derived neurons into five categories based on electrophysiological maturity, showed that cells with immature firing tended to have the lowest levels of synaptic activity, although whether RMP was also predictive was not addressed.

Several aspects of neuronal physiology showed significant developmental maturation in culture as well as genotypic differences between AS patients and control subjects. Resting membrane potential, which plays an important role in regulating neuronal excitability and the ability to fire APs, showed a developmental shift to a more hyperpolarizing potential that was not seen in AS-derived neurons. Although neurons from both AS and control subjects showed similar RMP values at the earliest time points, AS neurons remained significantly depolarized compared to controls at later times. AS-derived neurons were also different from controls with regard to the development of mature AP firing. Similar to RMP, no significant difference in AP firing between AS and control neurons was observed at the earliest time points. However, the proportion of neurons that fired mature APs or AP trains increased in control neurons at later times and was significantly greater than AS-derived neurons.

As observed with RMP and AP firing, AS neurons and control neurons showed similar levels of excitatory synaptic activity early in development, as measured by the percent of synaptically active neurons and frequency of spontaneous synaptic events. As development progressed, however, AS neurons reached a plateau in the percentage of cells that displayed spontaneous synaptic currents. Control neurons, on the other hand, continued to mature, reaching a significantly higher proportion of active neurons. Similarly, the frequency of synaptic events and the proportion of synaptically active neurons in AS and control cultures was not different early in their development, but by weeks 9–14, the proportion of active cells was significantly higher in control cultures compared to AS-derived cultures. Interestingly, there was also a small increase in synaptic event amplitude in AS neurons, possibly reflecting a compensatory response. The observed synaptic differences could be due to a number of factors including decreased release probability, fewer synaptic contacts or a larger proportion of silent synapses, that is, synapses that lack AMPA receptors. Further studies will be necessary to determine the contributions of these factors.

The connections between neurons are highly plastic, changing their size, strength and structure in response to a variety of stimuli^28^,^29^. Activity-dependent synaptic plasticity is known to be important for the proper development of neural circuits and may play a role in the pathophysiology of neurological disorders^14^,^30^,^31^. In fact, disrupted synaptic plasticity is a hallmark of AS mouse models^14^,^32^,^33^,^34^,^35^,^36^ and may underlie some of the cognitive impairments in AS patients. These plasticity deficits include disrupted hippocampal LTP, impairments in experience-dependent synaptic plasticity and impaired long-term depression (LTD). Impaired hippocampal LTP may underlie learning deficits in AS mice^35^ and other work has shown that this AS LTP phenotype may be due to changes in calcium/calmodulin-dependent protein kinase II^37^ and has been rescued with inhibition of the receptor tyrosine kinase ErbB4 (ref. 38). Interestingly, it seems that plasticity deficits may become more important with sensory experience during development, as dark rearing AS mice prevents LTP deficits^14^. Furthermore, type 5 metabotropic glutamate receptor-dependent LTD was shown to be enhanced in an AS mouse model^33^ and changes in cerebellar LTD have been associated with both loss of maternal *UBE3A* and duplication of *UBE3A* in mouse models^39^,^40^. Because of the relevance of these forms of plasticity to behavioural phenotypes in AS and other neurodevelopmental disorders, one of the critical challenges to the use of human stem cell models is generating neurons with the capacity to undergo activity-dependent plasticity^41^.

In the current study, we report the novel finding that iPSC-derived neurons exhibit NMDA receptor-dependent synaptic plasticity in culture. Both control and AS-derived neurons responded to the pharmacological induction protocol with large increases in activity, and though control neurons showed short-term (20–30 min) and long-term (50–60 min) synaptic plasticity expressed as an increase in the frequency of spontaneous synaptic events, long-term synaptic plasticity was significantly impaired in neurons from AS patients and *UBE3A* KO neurons compared to controls. The increase in synaptic current frequency with no change in amplitude suggests increases in presynaptic release probability and/or changes in synapse number due to formation of new synapses or insertion of AMPA receptors at silent synapses. This pharmacological LTP induction also caused long-term increases in AP firing in control neurons, as monitored by intracellular calcium imaging, but did not increase firing in AS neurons. Plasticity of AP firing has also been shown in human stem cell-derived neurons using multielectrode arrays^42^. Thus, this protocol provides a valuable model for more detailed investigations exploring differences in synaptic plasticity between AS and control neurons, including underlying molecular signalling pathways. Additionally, iPSC-derived neurons may provide insights into alterations in synaptic plasticity implicated in the pathophysiology of other neurogenetic disorders.

Stem cell-derived neurons have been increasingly used to identify pathophysiology in neurological disorders^43^,^44^,^45^,^46^,^47^. Previous studies of iPSC-derived neurons found that these cells can display repetitive AP firing, inward sodium currents >1 nA and input resistances consistent with the findings reported in this study^48^,^49^,^50^,^51^. Reported values for resting membrane potential and frequency of spontaneous synaptic currents are also similar^43^,^52^,^53^,^54^. The development of active and passive membrane properties as well as spontaneous synaptic activity has also been studied, although typically only up to 10–13 weeks *in vitro*^55^,^56^. Both of these previous studies report RMP, AP firing and levels of spontaneous synaptic activity that are similar to those observed in our cultures at the corresponding developmental time points. Interestingly, Devlin *et al*.^56^ shows no maturation of these properties over time, as the mean RMP, distribution of AP firing and proportion of neurons with spontaneous synaptic activity remains static throughout *in vitro* development. The Tang *et al*.^55^ study does show maturation of these properties from plating to 60 days post plating, and the findings in our study best match the maturation observed when astrocytes were included in their cultures, suggesting that our culture conditions provide an environment that supports continued maturation of these neuronal properties without requiring exogenous astrocytes or astrocyte-conditioned media. In support of this, we continue to see maturation of AP firing and half-width, transient outward currents, proportion of synaptically active neurons and frequency of spontaneous synaptic currents up to 20 weeks *in vitro*, which has not been reported in other studies. At our latest time points, most of our control neurons best match what Bardy *et al*.^27^ defines as Type 5 cells, in both level of synaptic activity and type of firing. Our AS-derived cultures seem to correspond to the more immature Type 1, Type 2 and Type 3 cells. Similar ranges of firing maturity as well as intrinsic currents and RMP have been observed in human embryonic stem cell-derived cultures^57^. Functional maturation in the present studies may be strongly enhanced by the presence of astrocytes intrinsic to the culture, as well as the addition of exogenous growth and survival factors, namely brain-derived neurotrophic factor and glial cell line-derived neurotrophic factor.

Because AS patients as well as mice with a maternally inherited *UBE3A* deletion show a seizure phenotype, changes in neuronal and/or synaptic properties that alter excitability are of great interest. It has been reported that neurons from AS model mice have abnormal spine morphology^13^ and decreases in the frequency of both spontaneous excitatory and inhibitory postsynaptic currents^9^. Supporting this finding, reduction of *UBE3A* expression via transfection with short hairpin RNAs that target *UBE3A* reduced AMPA receptor surface expression and decreased AMPA-mediated excitatory synaptic transmission in transfected neurons^7^. Interestingly, a study using mice with a maternally inherited deletion of *UBE3A* found that the RMP in hippocampal neurons from 8-week old animals was slightly hyperpolarized compared to controls^11^. Also, loss of Dube3a in *Drosophila melanogaster* leads to hyperpolarization in muscle cells^58^. The absence of a depolarized RMP in these models may be due to the difference in species or age at the time of recording. Overall, the similarities between findings in AS mouse models and the present results in human neurons suggest that patient-specific iPSC-derived neurons are capable of modelling deficits specific to the AS phenotype. In support of this, similar findings of altered excitatory synaptic transmission in a mouse model of Rett syndrome have also been observed in iPSC-derived neurons from Rett syndrome patients^45^,^59^.

In summary, the present study used iPSC-derived neurons from AS patients and unaffected control subjects to examine the maturation of neuronal and synaptic activity. In particular, our results reveal marked differences between AS-derived and control neurons in RMP and the development of intrinsic excitability, maturation of AP firing and excitatory synaptic activity and plasticity. These genotypic differences may provide a cellular phenotype for further investigations of the specific role of *UBE3A* and its downstream signalling mechanisms, and for identifying and evaluating therapeutic strategies to ameliorate the symptoms of AS and related neurodevelopmental pathologies.

## Methods

### Cell lines

iPSC lines from three AS patients (two males, one female) and four unrelated unaffected controls (three males, one female) were generated using either retroviral or lentiviral vectors expressing *OCT4*, *SOX2*, *KLF4*, *MYC* and *LIN28* (refs 60, 61). iPSC lines from two AS patients (one male and one female) harboured a large deletion of 15q11–q13. These iPSC lines and the female unaffected control were previously described and characterized^26^. The third AS line was generated from a male patient with a 2 bp deletion in *UBE3A*^62^ by the UConn-Wesleyan Stem Cell Core. Characterization of this iPSC line is shown in Supplementary Fig. 1a,b. Three control lines were generated from male donor subjects and have also been previously characterized^63^,^64^. For all cell lines, patient samples were obtained under appropriate Institutional Review Board protocols with consent. To disrupt *UBE3A* in a control iPSC line, CRISPR/Cas9 was used to KO *UBE3A* on both the maternal and paternal alleles. Small guide RNAs were designed to cut the *UBE3A* gene at the translational start site of protein isoform 1. As no template was provided for homology-directed repair, the error-prone non-homologous end-joining caused a 1 bp (G) insertion. Clones were screened by Sanger sequencing. This insertion disrupted splicing and/or translation resulting in the loss of *UBE3A* mRNA and protein. Characterization of this iPSC line is shown in Supplementary Fig. 1c.

### Cell culture

iPSCs were cultured on mitomycin C-treated mouse embryonic fibroblasts (Millipore) in conventional human embryonic stem cell medium consisting of DMEM-F12 (Life Technologies), 20% KO serum replacer, 0.1 mM non-essential amino acid (NEAA), 1 mM L-glutamine, 0.1 mM 2-mercaptoethanol and 10 ng ml^−1^ basic fibroblast growth factor. Cells were maintained in a humidified incubator at 37 °C with 5% CO_2_ and manually passaged once per week with the aid of a 28-gauge needle. Media were replaced daily. The protocol for neuronal differentiation was identical for control and AS iPSC lines and was adapted from Chamberlain *et al*.^26^. Briefly, iPSCs were manually detached and grown in suspension to form embryoid-like aggregates. The following day, cell aggregates were cultured in neural induction media (DMEM-F12, 1 × N2 Supplement, 0.1 mM NEAA, 2 μg ml^−1^ heparin and 10 ng ml^−1^ basic fibroblast growth factor). DMH1 (Millipore) and SB431542 (Stemgent) were added to the media at a final concentration of 2 μM on day 1 and day 4 of neural induction to enhance the formation of columnar cells^65^. The cell aggregates were then plated onto laminin-coated plates. Within a week, neural precursor cells were manually dissected and grown in suspension, forming neurospheres. After 6 days, the neurospheres were manually dissociated and plated in neural differentiation medium (Neurobasal, 1 × B-27 Supplement, 0.1 mM NEAA, 2 mM L-glutamine and 1 μg ml^−1^ laminin) onto polyornithine/laminin-coated glass coverslips. ROCK inhibitor Y-27,632 (10 μM; Wako) was added to the neural differentiation media during initial plating to promote cell attachment. After 2 days, 1 μM cAMP (Sigma), 0.2 mM L-ascorbic acid (Sigma), 20 ng ml^−1^ glial cell line-derived neurotrophic factor (Peprotech) and 20 ng ml^−1^ brain-derived neurotrophic factor (Peprotech) were added to the media. Media were replaced 2–3 times per week. Cells were grown in the absence of antibiotics.

ASOs against *UBE3A* and scrambled control ASOs were provided by Ionis Pharmaceuticals (Carlsbad, CA, USA). ASOs were synthesized as previously described^66^ and were 20 bp in length, with five 2′-*O*-methoxyethyl-modified nucleotides at each end of the oligonucleotide, ten DNA nucleotides in the centre and a phosphorothioate backbone. CNTL ASO, 5′-CCTTCCCTGAAGGTTCCTCC-3′; ASO-1, 5′-TGAGCTATCACCTATCCTTG-3′; ASO-2, 5′-CATTGTGATTTGTGTCCACT-3′. For treatment, coverslips of neurons were fed with fresh media and either ASO or scrambled control was immediately added to media at a concentration of 2.5 μM (low dose) or 10 μM (high dose) for ASO-1 or 8 μM for ASO-2 for 72 h, after which neurons were re-fed with fresh media. Electrophysiology experiments were carried out 3–6 weeks after the 72-h treatment.

Cell death was measured via an LDH Assay Kit (Takara) on control, AS, *UBE3A* KO and vehicle- and KCl-treated control cultures. Briefly, 600 μl of culture media was collected 72 h after feeding and then frozen at −20 °C. For control, AS and *UBE3A* KO cultures, media were collected twice from three coverslips during week 15 *in vitro* and then twice again at week 20 *in vitro*. For vehicle- and KCl-treated cultures, media were removed from eight vehicle-treated and eight KCl-treated coverslips once at 20+ weeks *in vitro*. Once all samples were collected, 50 μl of each sample was transferred to a 96-well plate where 50 μl of LDH reaction solution was added. LDH activity was measured by the intensity of red colour via a BMG Labtech (Ortenberg, Germany) CLARIOSTAR microplate reader at 490 nm. Plain feeding media and Triton-X-treated cultures were used as negative and positive controls, respectively.

### Electrophysiology

Whole-cell voltage and current clamp recordings were obtained from iPSC-derived neurons from AS patients and control subjects starting at 3 weeks post initiation of differentiation (3 days post plating). Neurons were identified based on morphology using infrared differential interference contrast video microscopy on an Olympus BX51W microscope. Individual coverslips were transferred to a recording chamber fixed to the stage of the microscope fitted with a × 40 water-immersion lens. The recording chamber was continuously perfused at ∼2 ml min^−1^ with oxygenated artificial cerebrospinal fluid (aCSF) (room temperature) containing (in mM) 125 NaCl, 2.5 KCl, 1.25 NaH_2_PO_4_, 2.0 MgCl_2_-6H_2_O, 25.0 NaHCO_3_, 15.0 dextrose and 2.0 CaCl_2_. Patch pipettes with resistances ranging from 3 to 5 MΩ were pulled from borosilicate glass capillaries with a Flaming/Brown P-97 micropipette puller (Sutter Instruments, Novato, CA, USA). Electrical recordings were made with an HEKA EPC10 amplifier (Heka Electronic, Darmstadt, Germany) and signals were filtered at 2.9 kHz and digitized at >6 kHz.

For current and voltage clamp recordings, pipettes were filled with an internal solution containing (in mM) 4.0 KCl, 125.0 K-gluconate, 10.0 HEPES, 10.0 phosphocreatine, 1.0 EGTA, 0.20 CaCl_2_, 4.0 Na_2_-ATP and 0.3 Na-GTP. Input resistance (*R*_i_) was monitored at the beginning and the end of each experiment by applying a 10 or 15 mV hyperpolarizing step. Neurons were rejected from analyses if (1) series resistance (*R*_s_) was >50 MΩ at the time of break-in, (2) *R*_i_ changed by >15% during the course of an experiment, or (3) *R*_i_ fell below 100 MΩ. On break-in, neurons were noted for their RMP by injection with 0 current. RMP values were corrected for the liquid junction potential calculated offline (JPcalc). AP firing was elicited in current-clamp mode by injecting current to hold the cells at ∼−70 mV, and applying 500 ms duration current steps from −20 to +40 pA in 5 pA intervals. Inward and outward currents were elicited by voltage-clamping cells at −70 mV and applying 300 ms steps from −100 to +40 mV in 10 mV increments. Spontaneous excitatory synaptic activity was monitored while holding the cell at −70 mV in voltage-clamp mode. For synaptic plasticity experiments, neurons were also held at −70 mV. Following a 10 min baseline recording in the presence of normal aCSF, aCSF supplemented with forskolin (50 μM; Tocris Bioscience), rolipram (0.1 μM; Tocris Bioscience) and 0 Mg^2+^ was perfused onto cells for 15 min, constituting the plasticity induction period^25^. Cells were then perfused with normal aCSF and held for up to 60 min post induction. Offline analysis was performed using Clampfit (Axon Instruments). Tests of statistical significance were conducted using analysis of variance, Student's *t*-test or the *χ*^2^ test as noted. Data are presented as mean±s.e.m.

### Calcium imaging

For all calcium imaging experiments, Fluo-4AM (Invitrogen) was mixed with 10 μl Powerload (Invitrogen) and vortexed for 15 s. Fluo-4AM/Powerload mix was subsequently mixed with aCSF for a final concentration of 10 μM Fluo-4AM. Individual coverslips were removed from culture media and incubated in 600 μl of Fluo-4AM mixture for 1 h at 37 °C. Following incubation, coverslips were placed in a recording chamber on an Olympus BX51W microscope and rinsed for 15 min with continuous flow of aCSF before imaging. Cells were imaged at 470 nM (Cairn OptoLED). Areas of the coverslip with >10 healthy looking cells with neuronal morphology were chosen for imaging. Calcium signals were acquired using Turbo-SM software and an SM-CCD67 camera (RedShirtImaging, Decatur, GA, USA). For spontaneous activity, images were acquired for 6 min at 100 Hz. For plasticity experiments, images were acquired for 90 min (see plasticity protocol above) at 10 Hz. Following data acquisition, all experiments were randomized and coded and then analysed by a blinded experimenter. Cells with neuronal morphologies were chosen as regions of interest across all experiments. Traces were then blindly analysed offline in Clampfit (Axon Instruments). For 100 Hz experiments, transients were counted if they had rise times ≤15 s. For 10 Hz experiments, transients were counted if they had rise times ≤120 s.

### Immunocytochemistry

iPSC-derived neurons were fixed in 4% paraformaldehyde in PBS for 15 min. Cells were permeabilized in 0.1% Triton X-100, 10% goat serum and PBS for 10 min. Cells were then blocked in 10% goat serum in PBS for 30 min, and incubated overnight at 4 °C in primary antibodies against TUJ1 (mouse monoclonal, 1:200; Covance or chicken polyclonal, 1:500; Abcam), and either TBR1 (rabbit polyclonal, 1:1,000; Proteintech), GAD67 (mouse monoclonal, 1:1,000; Abcam), S100β (rabbit monoclonal, 1:200; Abcam), CTIP2 (rat monoclonal, 1:500; Abcam), SATB2 (mouse monoclonal, 1:50; Abcam), CUX1 (CDP, rabbit polyclonal, 1:50; Santa Cruz) or tyrosine hydroxylase (mouse monoclonal, 1:200; Millipore). Cells were then incubated in darkness with respective secondary antibodies Alexa Fluor 488 (goat anti-mouse, 1:200; Life Technologies), and either Alexa Fluor 594 (goat anti-rabbit or goat ant-rat, 1:200; Life Technologies) and/or Alexa Fluor 647 (goat anti-chicken; Life Technologies) for 1 h. Nuclei were counterstained with DAPI (H-1,500; Vector Labs). Images were captured with a Zeiss Axiovision fluorescence microscope. For quantification, at least four fields per coverslip were randomly selected and the number of cells that immunostained positive for specific markers were counted by individuals blind to the experimental conditions. iPSCs from AS patient 3 and from the isogenic *UBE3A* KO line were validated for pluripotency using immunocytochemistry (Supplementary Fig. 1). The following antibodies and concentrations were used: OCT3/4 (mouse monoclonal, 1:200; Santa Cruz), TRA-1–60 (mouse monoclonal, 1:200; Santa Cruz) and SSEA-4 (mouse monoclonal, 1:20; Developmental Studies Hybridoma Bank). Nuclei were counterstained with DAPI.

### Flow cytometry

Cells were collected, fixed in 0.1% paraformaldehyde or ice-cold methanol, and then permeabilized. The cells were incubated overnight with primary antibodies, followed by fluorophore-conjugated secondary antibodies for 1 h. Cell nuclei were counterstained with DAPI. Cells were analysed on BD LSR II, using the BD FACS DIVA analysis software. A minimum of 10,000 DAPI+ cells were collected. Cells were gated on single nucleated cells and quadrant gates were created based on unstained cells (no antibodies added). Images of the plots used to set the gates are shown in Supplementary Fig. 2.

### Western blots and quantitative PCR

Neurons were lysed in Cell Lysis Buffer (Cell Signaling Technology) supplemented with Protease Inhibitor Cocktail II (Calbiochem) and total protein concentration was quantified using the BCA Protein Assay Kit (ThermoFisher). Twenty-five micrograms of each total neuron lysate was separated by SDS–polyacrylamide gel electrophoresis on a 4–20% TGX mini-gel (Bio-Rad). Protein was transferred to PVDF membranes using the TransBlot Turbo system (Bio-Rad). Membranes were blocked for 1 h at room temperature in 5% non-fat milk in TBS-T (Tris-buffered saline with 0.1% Tween-20) before incubation in primary antibody diluted in blocking buffer overnight at 4 °C. Washes were performed in TBS-T at room temperature for 10 min each. Membranes were then incubated in secondary antibody at room temperature for 1 h. The following antibodies were used: mouse anti-GAPDH (Millipore, 1:2,000), mouse anti-PSD-95 (NeuroMabs, 1:1,000), mouse anti-UBE3A (Bethyl Laboratories Inc., 1:1,000 or Becton Dickinson, 1:250), horseradish peroxidase -conjugated goat anti-mouse secondary antibody (ThermoFisher, 1:3,000). Western blots were detected using Clarity Western ECL substrate (Bio-Rad) and imaged with the ChemiDoc Touch Imaging System (Bio-Rad). Images of cropped and uncropped Western blots with molecular weight markers are shown in Supplementary Fig. 3f,g. Quantitative PCR for pluripotency markers and for changes in *UBE3A* via ASOs or topotecan was performed as previously described^47^. Briefly, total RNA was isolated using RNA-Bee (AMS Biotechnology) according to the manufacturer's protocol. cDNA was produced using the High Capacity cDNA Reverse Transcription Kit (Life Technologies). Assays used were TaqMan Gene Expression Assays (Life Technologies). Expression levels for pluripotency genes were normalized to the housekeeping gene *GAPDH*. Expression levels for *UBE3A* via ASOs or topotecan were normalized to scramble or vehicle levels, respectively.

### Data availability

The data that support the findings of this study are available from the corresponding author on reasonable request.

## Additional information

**How to cite this article:** Fink, J. J. *et al*. Disrupted neuronal maturation in Angelman syndrome-derived induced pluripotent stem cells. *Nat. Commun.* **8,** 15038 doi: 10.1038/ncomms15038 (2017).

**Publisher's note:** Springer Nature remains neutral with regard to jurisdictional claims in published maps and institutional affiliations.

## Supplementary Material

Supplementary Information Supplementary Figures
