# Supplementary material for: Disrupted neuronal maturation in Angelman syndrome-derived induced pluripotent stem cells
Source: Nat Commun. 2017 Apr 24;8:15038. doi: 10.1038/ncomms15038 (PMC5413969; doi:10.1038/ncomms15038)
Supplement: Supplementary Information — Supplementary Figures [file ncomms15038-s1.pdf]

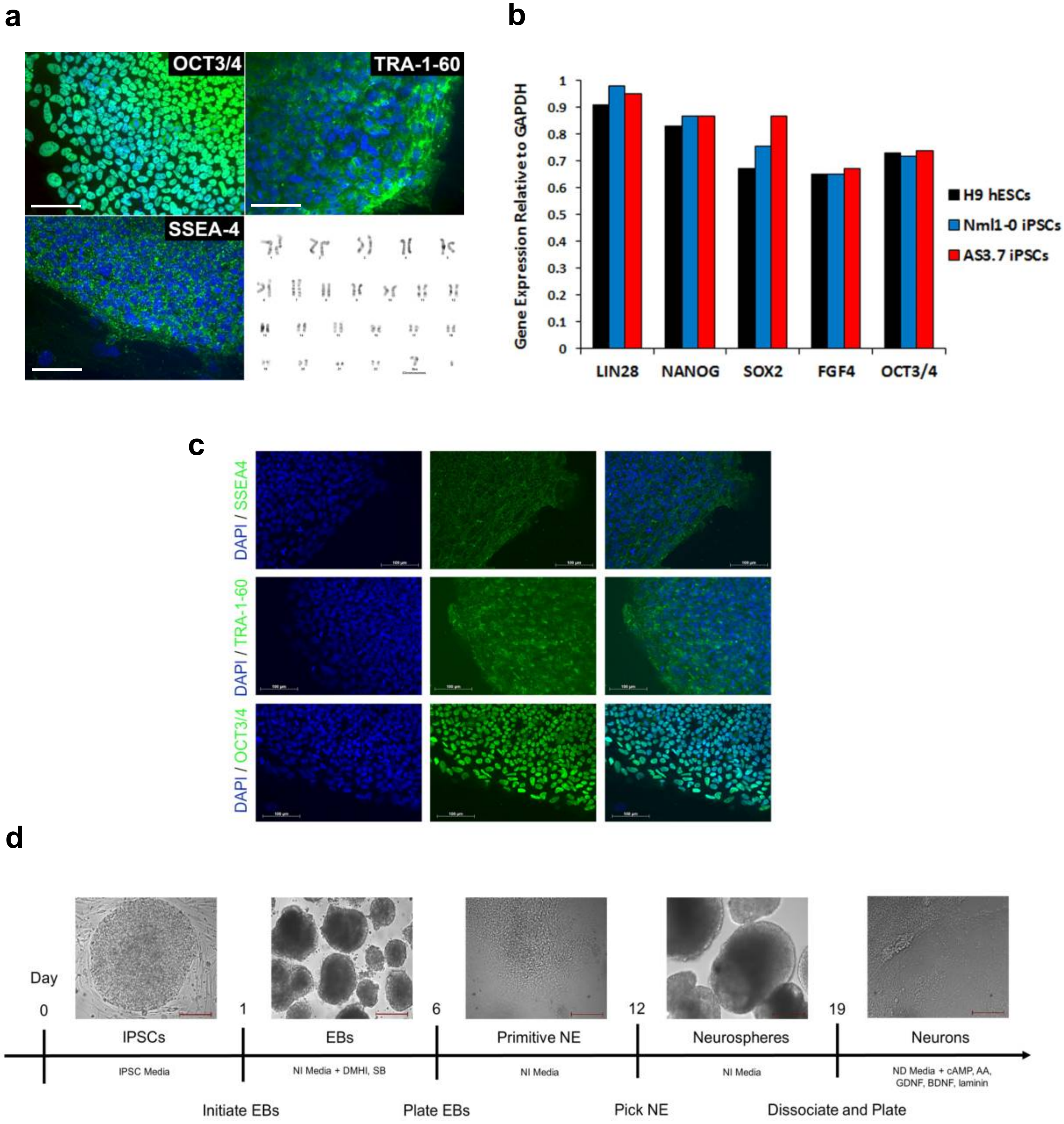

Supplementary Fig. 1

Supplementary Figure 1. Characterization of induced pluripotent stem cells (iPSCs) and differentiation protocol. (**a**) Immnocytochemistry for the pluripotency markers OCT3/4, TRA-1-60, and SSEA-4 in iPSCs from AS patient 3, a previously unpublished cell line. Nuclei are counterstained with DAPI (blue). Scale bar: 100 µm. Lower right panel depicts karyogram for this line. (**b**) Quantification of gene expression for pluripotency markers in iPSCs derived from AS patient 3. (**c**) iPSCs from isogenic *UBE3A* knockout line stained for pluripotency markers. (**d**) Diagrammatic representation and representative phase contrast images of neuronal differentiation protocol. EB, embryoid body; NE, neuroepithelium.

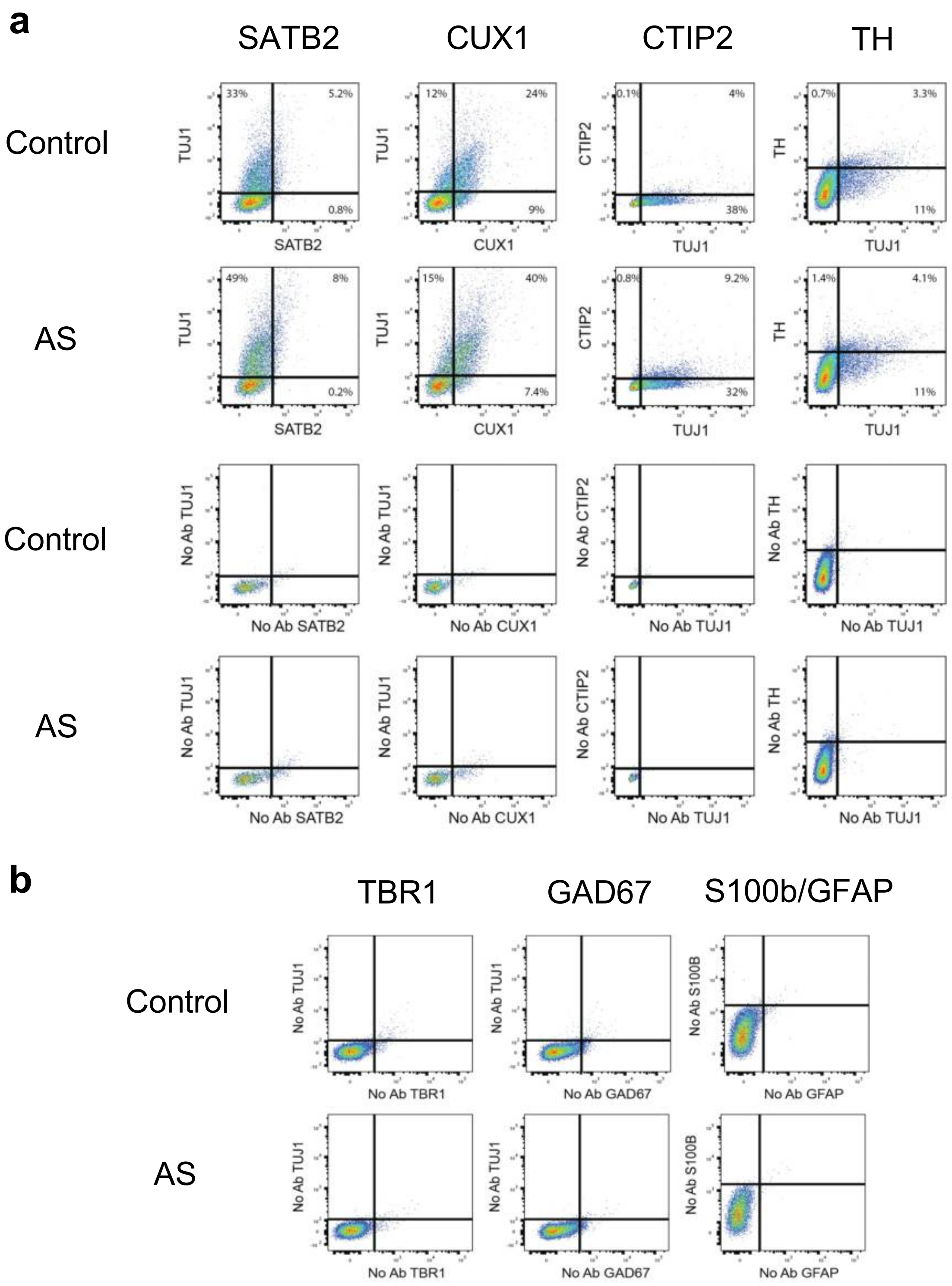

Supplementary Fig. 2

Supplementary Figure 2. Flow analysis for cell-type specific markers. (**a**) Flow cytometry graphs for SATB2/TUJ1, CTIP2/TUJ1, CUX1/TUJ1, and tyrosine hydroxylase (TH) /TUJ1 in control and AS-derived cultures. Experiments were carried out between 10 - 22 weeks *in vitro*. For each cell marker, matching AS and control experiments were done at similar ages. Below are images of the plots used to set the gates. Cells were gated on single nucleated cells and quadrant gates were created based on unstained cells (no antibodies added). (**b**) Images of the plots used to set the gates for data shown in Fig. 1b.

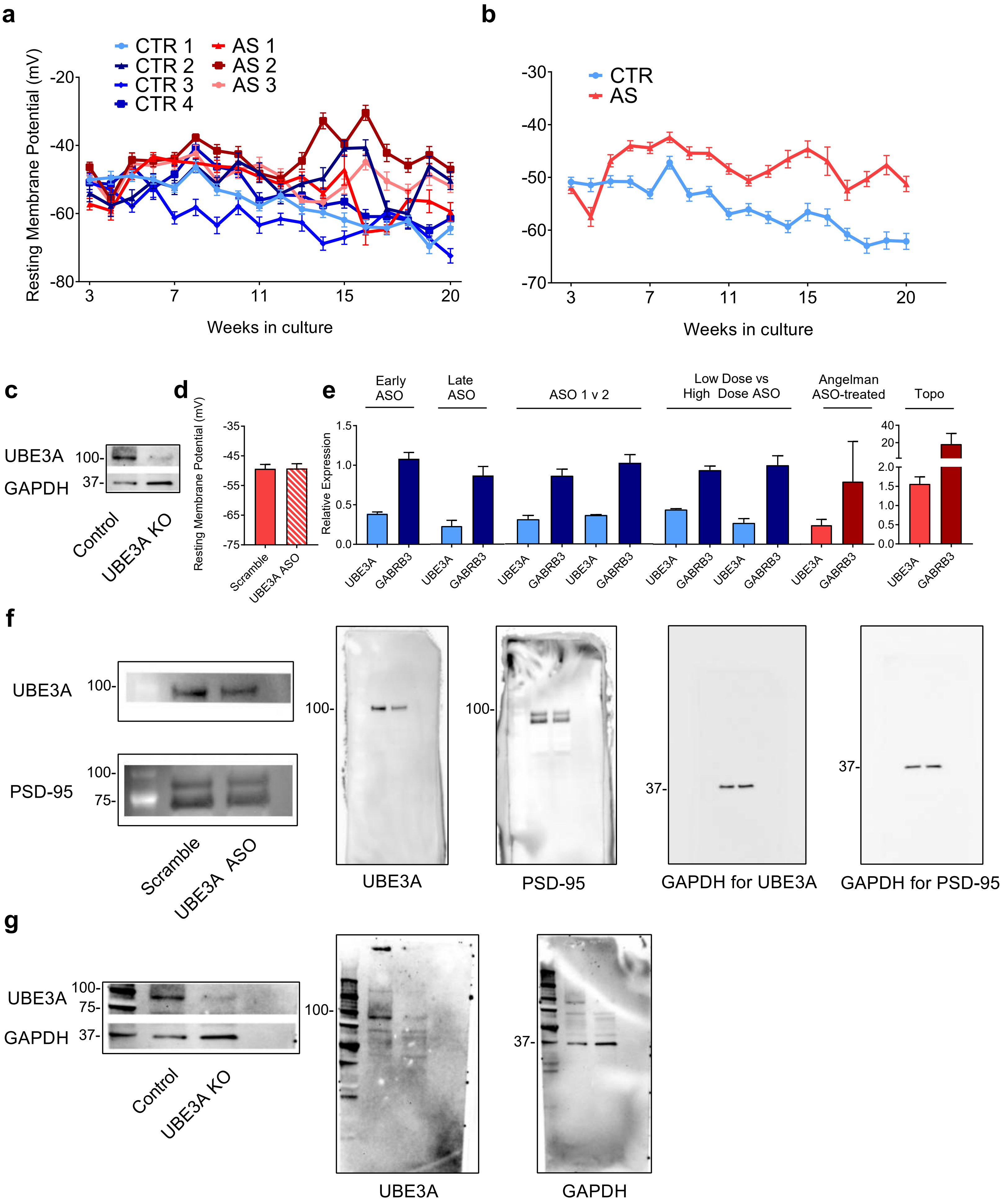

Supplementary Fig. 3

Supplementary Fig. 3. Additional resting membrane potential (RMP) analysis. (**a**) RMP data for individual patients at all weeks. n>15 for all lines. (**b**) Group data for RMP of CTR and AS-derived neurons during development; n≥60 for CTR at each week, n>45 for AS at each week. (**c**) Western blot of UBE3A (100 kD) and GAPDH (37 kD) levels in induced pluripotent stem cells from *UBE3A* knockout line. (**d**) Resting membrane potential of AS patient 3 treated with *UBE3A* antisense oligonucleotides (ASOs). (**e**) *UBE3A* and *GABARB3* mRNA expression (normalized to scramble levels) via PCR for (from left to right) control neurons treated with *UBE3A* ASO at 6 weeks in culture (6 coverslips/condition, 15 cells/coverslip), control neurons treated with *UBE3A* ASO at 18 weeks in culture (4 coverslips/condition, 15 cells/coverslip), control neurons treated with UBE3A ASO 1 vs treatment with UBE3A ASO 2 (n=15 cells/coverslip for 8 coverslips of both scramble and ASO 1 and 2 coverslips for both scramble and ASO 2), control neurons treated with low dose (2.5 µM) ASO (4 coverslips/condition, 15 cells/coverslip) and high dose (10 µM) ASO (6 coverslips/condition, 15 cells/coverslip), AS neurons treated with *UBE3A* ASO (n=15 cells/coverslip for 2 coverslips for both scramble and ASO), AS neurons treated with topotecan (1 µM; n=15 cells/coverslip for 2 coverslips for vehicle and 4 coverslips for topotecan). (**f**,**g**) Cropped (left) and uncropped (right) Western blots with molecular weight markers for data presented in Fig. 2d (**f**) and Supp. Fig. 3c (**g**).

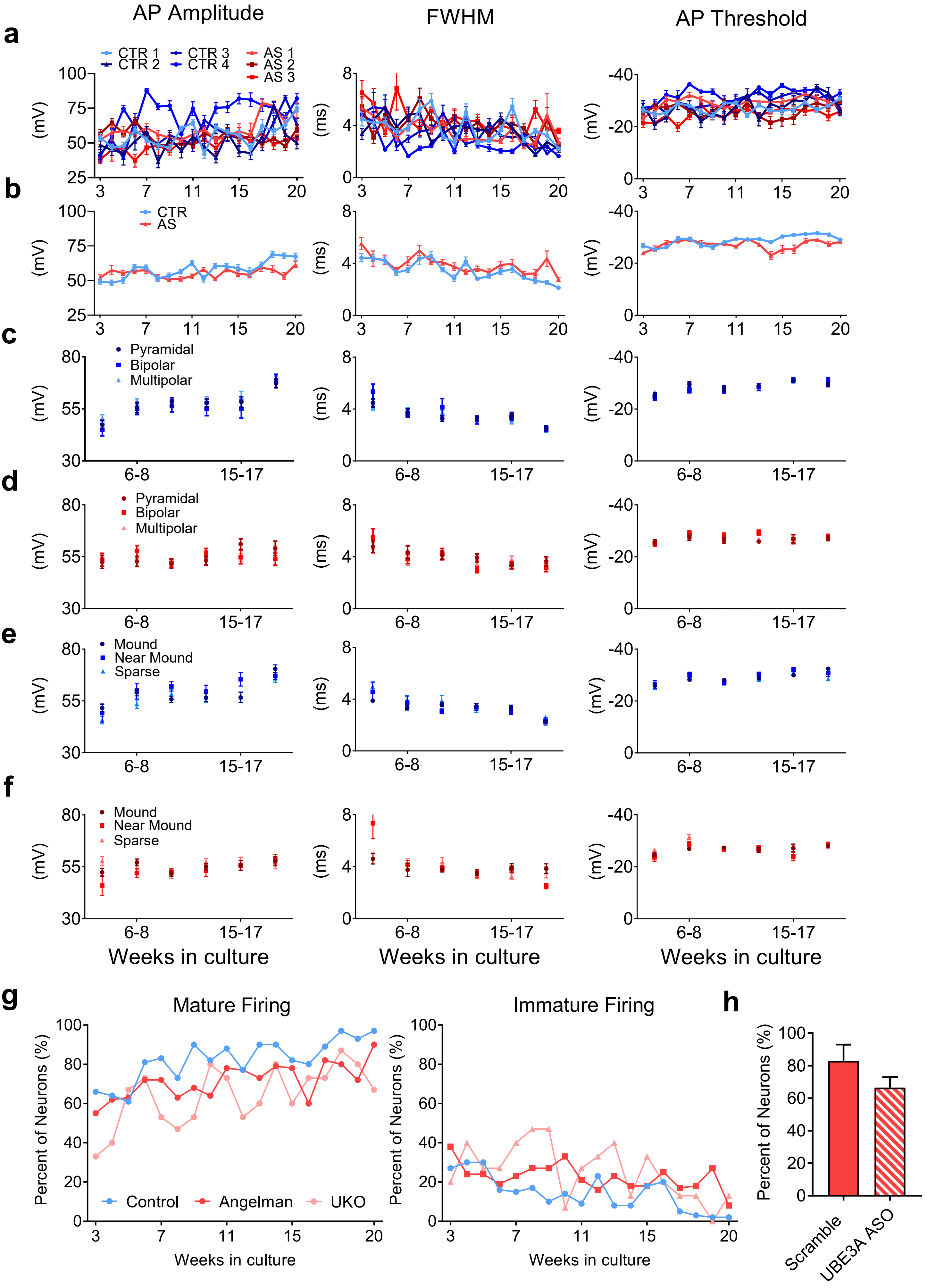

Supplementary Fig. 4

Supplementary Fig. 4. Additional action potential (AP) feature analysis. (**a-f**) AP amplitude (left), full-width at half-maximal amplitude (FWHM; middle), and AP threshold (right) for (**a**) individual control (CTR) and Angelman syndrome (AS) subjects at all developmental weeks. At all weeks, n>8 for all lines. (**b**) Group data for CTR and AS neurons at all developmental weeks. For control, each symbol represents data from 4 subjects. For AS, each symbol represents data from 3 subjects (n>40 cells for each week). (**c, d**). Group data for CTR and AS neurons by morphology. (**e, f**) Group data for CTR and AS neurons by location. For controls, each symbol represents data from 4 subjects; For AS, each symbol represents data from 3 subjects (n>25 at each symbol for both genotypes for all locations and morphologies). (**g**) Percentage of cells showing mature firing (either single mature or mature train; left) or immature firing (right) week by week across all 20 weeks of *in vitro* development (n>40 for all genotypes at all weeks). (**h**) Percent of cells showing mature firing from cultures derived from AS patient 3 treated with *UBE3A* ASO (n=15 cells/coverslip for 2 coverslips of both scramble and ASO treated cultures).

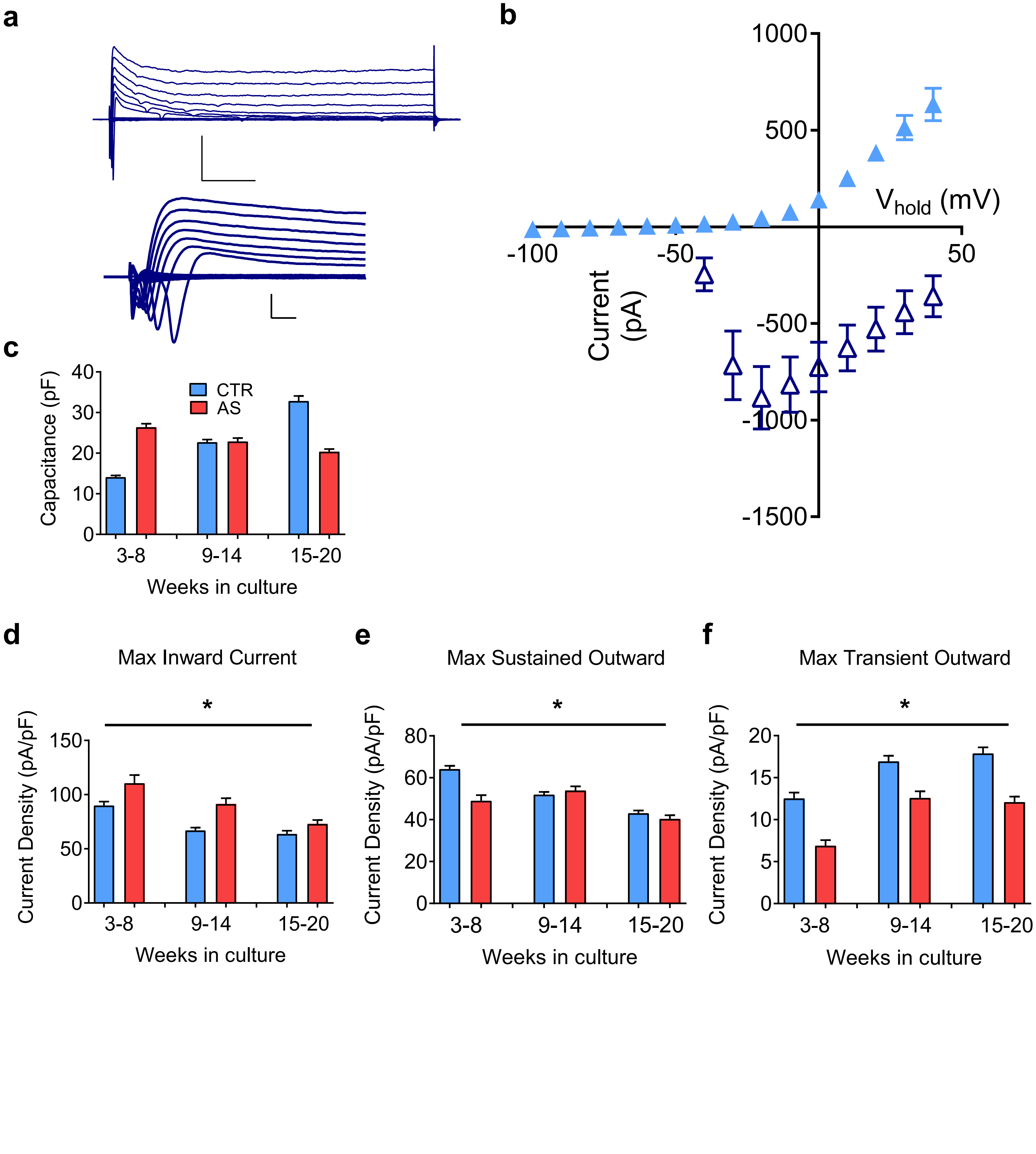

Supplementary Fig. 5

Supplementary Fig. 5. Intrinsic membrane current analysis. (**a**) Top: Example traces from a whole-cell recording of a control neuron at week 12 in culture. Scale bar: 1 nA, 50 ms. Bottom: Same traces as above on an expanded time scale. Scale bar: 500 pA, 2 ms. (**b**) Current-voltage relationship of inward and sustained outward currents measured from control neurons at 12 weeks in culture (n=14). (**c-f**) Group data for capacitance (**c**) inward current density (**d**), sustained outward current density (**e**), and transient outward current density (**f**) for neurons derived from both control and AS subjects during *in vitro* development. For control, each bar represents data from 4 subjects. For AS, each bar represents data from 3 subjects (n>250 for both genotypes at all 3 time periods). *, $p < 0.0001$, 2-way ANOVA.

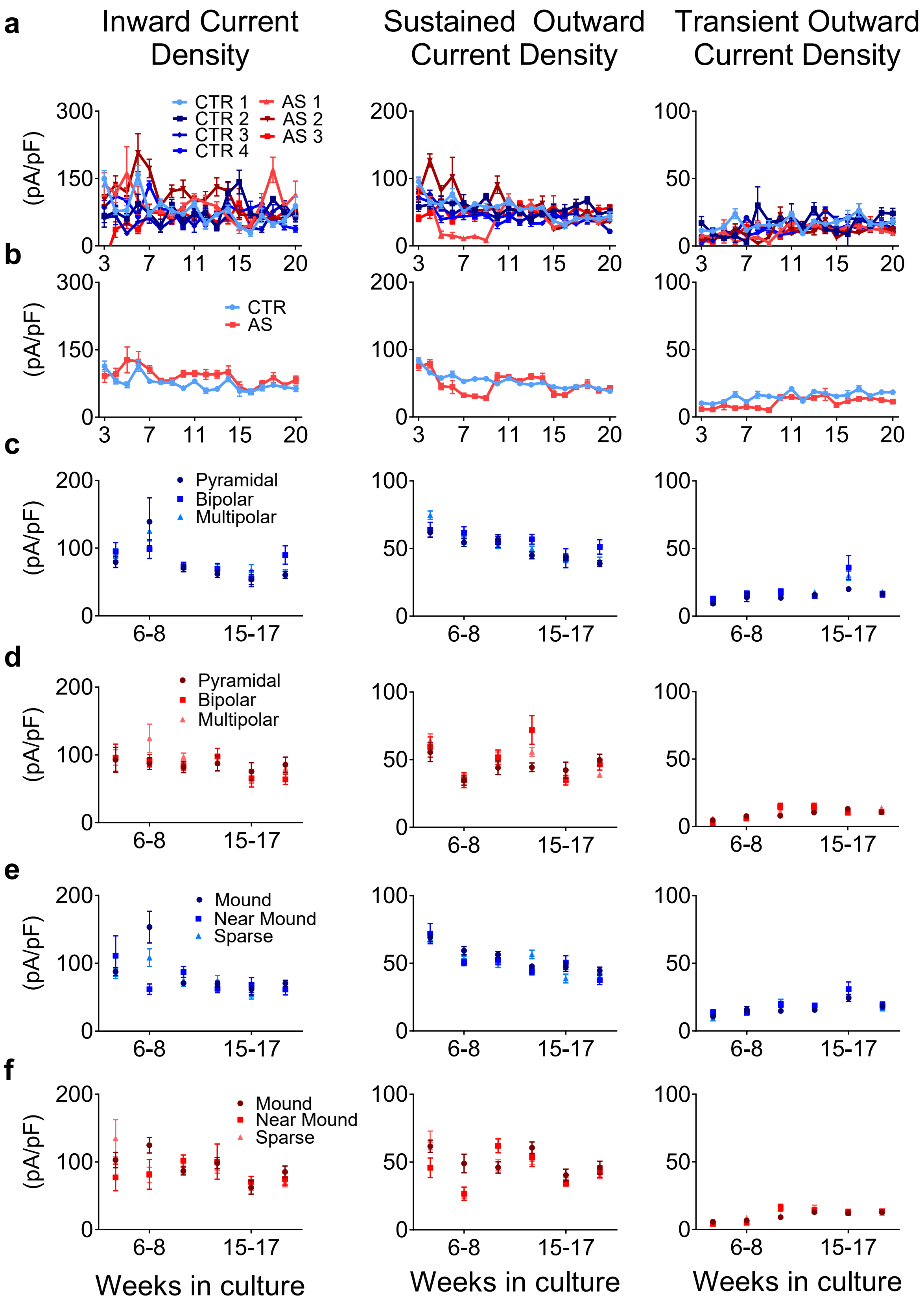

Supplementary Fig. 6

Supplementary Fig. 6. Additional intrinsic membrane current analysis. (**a-f**) Maximum inward current density (left), maximum sustained outward current density (middle), and maximum transient current density (right) for (**a**) individual control (CTR) and Angelman syndrome (AS) subjects at all weeks (n>10 per week). (**b**) Group data for CTR and AS subjects at all weeks (n>40 for both genotypes at every time point). (**c, d**) Group data for CTR and AS neurons by morphology (n>20 for all morphologies at every time point) (**e, f**) Group data for CTR and AS neurons by location (n>20). (**b-f**) For control, each symbol represents data from 4 subjects. For AS, each symbol represents data from 3 subjects.

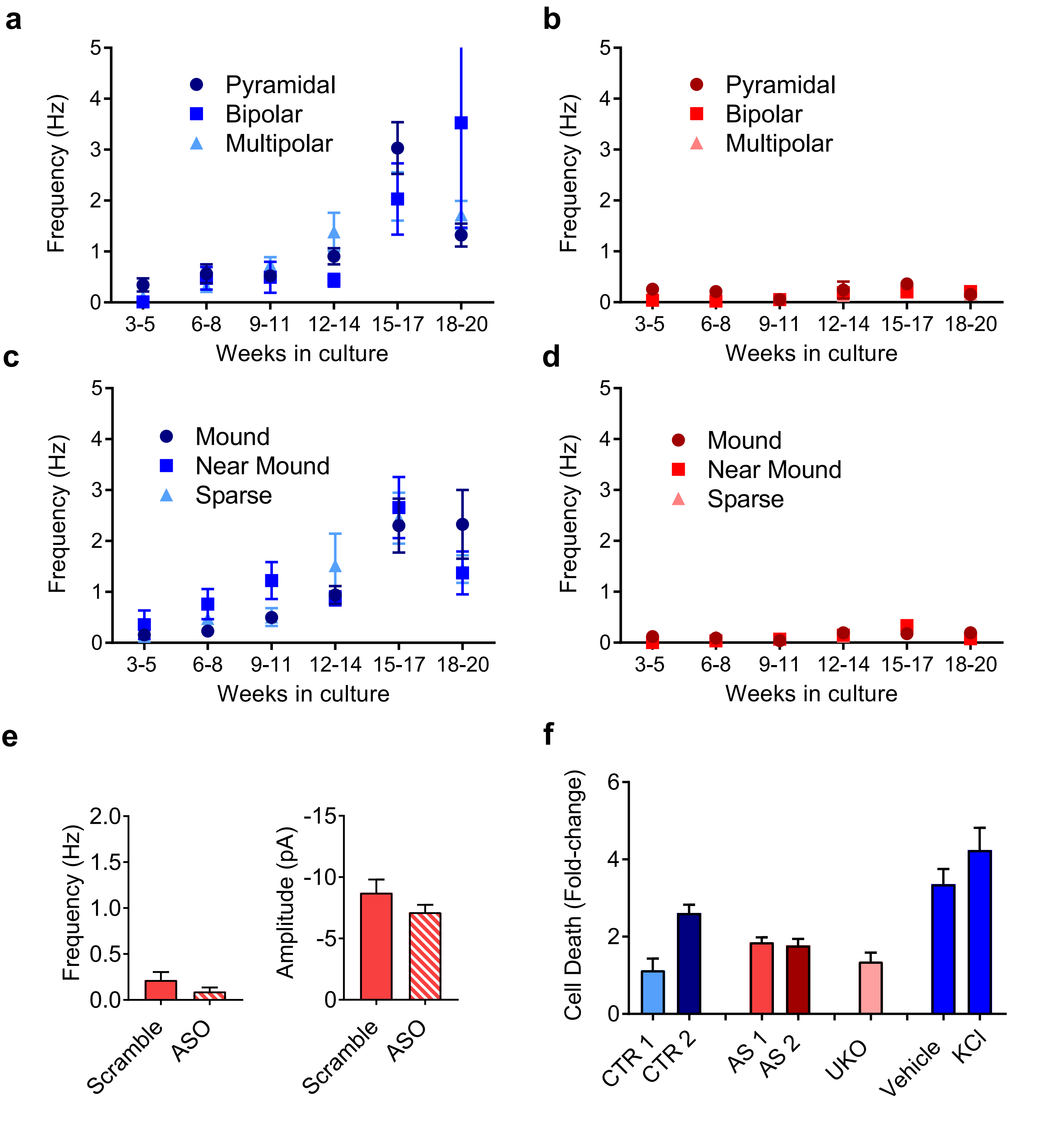

Supplementary Fig. 7

Supplementary Fig. 7. Additional synaptic activity analysis. (**a-b**) Frequency of spontaneous synaptic currents for (**a**) control (CTR) neurons and (**b**) Angelman syndrome (AS) neurons separated by morphology. (**c, d**) Frequency of spontaneous synaptic currents for (**c**) CTR and (**d**) AS neurons separated by location. For control, each symbol represents data from 4 subjects. For AS, each symbol represents data from 3 subjects (n>20 for all time points). (**e**) Spontaneous synaptic frequency (left) and event amplitude (right) for cultures derived from AS patient 3 treated with *UBE3A* ASO (2 coverslips, 15 cells/coverslip). (**f**) Cell death as measured by LDH activity (normalized to plain culture media) for 2 CTR lines (6 samples/condition/line), 2 AS lines (6 samples/condition/line), and a UBE3A KO line (3 samples/condition/line) as well as vehicle- and KCl-treated cultures (2 samples/condition x 4 lines).
